# Supplementary material for: Regional variations in child health deprivation and its associated factors in Nigeria
Source: PLOS Glob Public Health. 2025 Aug 1;5(8):e0003796. doi: 10.1371/journal.pgph.0003796 (PMC12316245; doi:10.1371/journal.pgph.0003796)
Supplement: Attachment — Submitted filename: PLOS Manuscript 1.pdf [file pgph.0003796.s001.pdf]

Regional variations in child health deprivation and its associated factors in Nigeria - Manuscript

I appreciate the opportunity to evaluate this manuscript. These are significant concerns, and I think that fixing them will improve the article's quality and acceptability for publishing. They are listed in the following order:

Abstract: The authors should include a brief explanation of the specific child health indicators used in the, such as child's age, sex, education of the household head, and household size, to provide more context for the study's focus, while also highlighting the regional variations examined. Listing the various descriptive statistical tools used in the analysis is irrelevant and can be simplified to state that "the data were analyzed using different descriptive statistics to examine regional variations in child health." Furthermore, since the study relies on secondary data, explaining the study design is unnecessary in the abstract. Instead, the authors should summarize the relevance of the results using statistical tests such as Pearson Chi-square and Binary Logistic Regression to strengthen the methodological accuracy. Additionally, the results section of the abstract need improvement, as it does not adequately report the findings, and the percentage prevalence of child health deprivation mentioned is not found in the methodology. Lastly, the authors should discuss potential policy recommendations or future research directions based on the results, and since diarrhea and fever were not included as variables in the analysis, they should not be mentioned in the conclusion regarding treatment services.

Introduction: The introduction provides a strong foundation for the research. It effectively highlights the prevalence of child poverty and its impact especially focusing on child development, both globally and in Nigeria. Furthermore, the use of previous statistical results helps emphasize the gravity of the problem, making the argument more compelling. The authors clearly define "child deprivation" and its various dimensions, which enhances understanding. The introduction is generally well-structured, transitioning smoothly from global to regional issues. However, there are a few suggestions for improvement;

The introduction is overly lengthy and contains several wordy or repetitive sentences, likely for emphasis. Additionally, there are inconsistencies in the use of terms such as "child health deprivation," "child deprivation," "health deprivation," "children under 5," and "under-five children," which can confuse the reader. It is important to stick to one consistent term throughout the introduction for clarity. Condensing the information by paraphrasing or summarizing instead of quoting would also help streamline the text.

The connection between child poverty and health deprivation needs to be further strengthened. Although this was briefly discussed in the abstract, it would be more effective to elaborate on it by citing specific studies or statistics that show the direct impact of poverty on child health outcomes.

Additionally, there is inconsistency in the statistics reported regarding child poverty trends in line "4" and "6" where the authors wrote "a drop from 385 million in 2022" "to 333 million in 2022" appears to have an error. The second figure should likely refer to a different year, so this should be corrected for clarity. The statement about the reduction in child poverty from 50.2% to 43.4% is

also somewhat repetitive, as the previous sentence already discusses trends. Consider merging these points into a single, concise statement to reduce redundancy and improve flow.

Literature: The definitions provided for child deprivation by UNICEF and CFI are helpful but slightly broad, relying on immunization, treatment for diarrhea, and skilled birth attendance.

However, the study lacks a nuanced discussion of broader definitions of child health deprivation. The second definition from CFI could benefit from a clearer transition between the three key concepts—deprivation, exclusion, and vulnerability—by streamlining the definitions and providing brief, clear explanations for each term. The authors should consider expanding the operationalization of child health deprivation by discussing it in terms of access to healthcare, nutrition, sanitation, or psychosocial well-being. For instance, WHO and UNICEF use a multidimensional approach to child deprivation that includes nutrition, education, and living conditions.

The review briefly explain socio-economic factors, household size, and family characteristics but does not adequately discuss other important drivers, such as maternal education, geographic barriers to healthcare, and cultural factors. The authors should explore how parental education, access to maternal health services, cultural beliefs about health, and regional healthcare infrastructure contribute to child health deprivation. Additionally, the role of gender inequality in healthcare access, which disproportionately affects children, particularly girls, in some regions, should also be considered.

While the review mentioned urbanization, it does not fully address the urban-rural health divide, especially in relation to urban poverty and slums, which significantly contribute to child health deprivation. The authors should expand on urban-rural disparities by citing literature on how urbanization exacerbates inequalities in healthcare access, particularly in rapidly growing slum populations. Studies from African countries could illustrate the nuances of urban poverty compared to rural deprivation, including the environmental risks in slum areas, such as pollution, poor sanitation, and unsafe drinking water, which need more thorough discussion.

Although the review focuses on Nigeria, it misses the opportunity to place the country's child health deprivation in a global context. The authors should include comparative studies from other developing regions, such as South Asia or Latin America, facing similar challenges. They could discuss whether Nigeria's child health deprivation patterns mirror or diverge from those in other African countries or developing regions. Recent global reports from organizations like the World Bank, WHO, or UNICEF could provide valuable comparative data, framing Nigeria’s situation within broader global health trends.

The transition from global data to Nigeria-specific information could be improved by adding a sentence or two that explicitly connects global trends in child health deprivation to Nigeria’s context before delving into local data. The introduction of the discussion on health insurance seems abrupt and would benefit from a clearer link to child health deprivation. The authors should first explain how healthcare access impacts child health deprivation before introducing the state of Nigeria’s health insurance system. Minor grammatical improvements, such as changing "present a need to probe" to "indicate the need to investigate," could enhance readability.

Finally, although regional disparities in child health deprivation are mentioned, this important theme gets overshadowed by general discussions of deprivation. The concluding sentence is too long and lacks specificity about how the authors' findings will contribute to addressing child health deprivation. Clarifying these points would strengthen the conclusion.

Methodology: The methodology section is thorough, but there are a few areas that could benefit from clarification, restructuring, and some technical adjustments. Here are specific recommendations for improvement.

The details about the MICS 2021 survey and its sampling frame are repeated in both the survey design and data source sections, which could be streamlined for clarity. There is confusion between the original sample size (31,103) and the final sample size (26,639) after weighting, but no explanation is given for this discrepancy. Additionally, the methodology lacks mention of the software or statistical techniques used for data analysis.

While the description of the statistical techniques is thorough, it is somewhat verbose and could be more concise. The rationale for choosing specific statistical methods is not clearly explained. Though the data cleaning process is mentioned, it lacks sufficient detail on the steps taken to check for errors.

The results for the parameters of the binary logistic regression are not included, and the goodness-of-fit measure for the logistic regression model, derived from the likelihood ratio test, is missing. This measure is important as it indicates how well the model predicts the outcome variable. Consequently, the methods used to assess the overall performance of the fitted binary logistic regression model are also omitted. Streamlining these aspects and providing necessary details on data analysis would improve the clarity and completeness of the methodology.

Results: Tables 1, 2, 3, and 4 are missing. As mentioned earlier in the preceding paragraph, some statistical results are not included. It is also very important to check for the presence or absence of multicollinearity before analyzing the data using binary logistic regression which is missing in this study. The results of the findings the authors presented does not capture the research questions. There is need for more statistical analysis that would further strengthen the research study.

Discussion: The discussion section of the study does a commendable job of linking the findings with previous literature, but there are a few gaps that could be addressed for a more comprehensive analysis. Below are the missing points and areas that could benefit from further elaboration.

While the authors acknowledge the limitations of using secondary data, they did not adequately discuss how this affects the validity or generalizability of the findings. For example, the results may be skewed due to underreporting in certain regions or among certain demographic groups? Providing a more detailed discussion of how secondary data limitations could have influenced key findings and offering suggestions for future studies using primary data would enhance the rigor of the analysis.

References: The references appear to be well-written and relevant to the topic of child health deprivation in Nigeria. However, there seems to be a duplicate entry for the Nigeria Multiple Indicator Cluster Survey (MICS) (references 17 & 18). The authors might want to confirm if they are indeed different reports. Also consider including geographically relevant resources e.g., while the focus is on Nigeria, some references on child health in neighboring countries or similar contexts might be insightful for comparison.
