# Supplementary material for: Regional variations in child health deprivation and its associated factors in Nigeria
Source: PLOS Glob Public Health. 2025 Aug 1;5(8):e0003796. doi: 10.1371/journal.pgph.0003796 (PMC12316245; doi:10.1371/journal.pgph.0003796)
Supplement: Attachment — Submitted filename: Response to Reviewers.docx [file pgph.0003796.s003.docx]

Academic Editor,
PLOS Global Public Health

**Rebuttal letter on Regional variations in child health deprivation and its associated factors in Nigeria – Manuscript**

We thank you for giving us the opportunity to strengthen this manuscript with your valuable comments and queries. We have worked hard to incorporate your feedback and hope that these revisions persuade you to accept our submission. Our responses are as follows:

**Journal Requirements**

**Comment 1:** Please note that your Data Availability Statement is currently missing [the repository name and/or the DOI/accession number of each dataset OR a direct link to access each database]. If your manuscript is accepted for publication, you will be asked to provide these details on a very short timeline. We therefore suggest that you provide this information now, though we will not hold up the peer review process if you are unable.

**Response: Thank you. Data Availability Statement has been provided. The datasets are available online at** [**https://mics.unicef.org/surveys**](https://mics.unicef.org/surveys)

**Comment 2:** Please provide separate figure files in .tif or .eps format.

**Response: Thank you. Separate figure files in .tif format has been provided.**

**Comment 3:** Tables should not be uploaded as individual files. Please remove these files and include the Tables in your manuscript file as editable, cell-based objects.

**Response: Thank you.** Tables have been uploaded in the manuscript file and as editable, cell-based objects.

**Review Comments to the Author**

**Reviewer:** I appreciate the opportunity to evaluate this manuscript. These are significant concerns, and I think that fixing them will improve the article's quality and acceptability for publishing. They are listed in the following order:

**Response: Thank you for the time spent in reviewing this manuscript.**

**Abstract:**

**Comment:** The authors should include a brief explanation of the specific child health indicators used in the, such as child’s age, sex, education of the household head, and household size, to provide more context for the study's focus, while also highlighting the regional variations examined.

**Response: Thank you for your suggestion. We have incorporated this suggestion and provide more context for the study's focus. The regional variations examined in the manuscript has been presented.**

**Comment:** Listing the various descriptive statistical tools used in the analysis is irrelevant and can be simplified to state that "the data were analysed using different descriptive statistics to examine regional variations in child health."

**Response: Thank you. We have incorporated your comments. It now read: “Data were analysed using different descriptive statistics to examine regional variations in child health deprivation. Pearson Chi-square statistical test and binary logistic regression model were performed to determine associated factors influencing child health deprivation in Nigeria at p-value of less than 0.05 level of significance”.**

**Comment:** Furthermore, since the study relies on secondary data, explaining the study design is unnecessary in the abstract. Instead, the authors should summarize the relevance of the results using statistical tests such as Pearson Chi-square and Binary Logistic Regression to strengthen the methodological accuracy.

**Response: Thank you. Adjustment has been made. The study design has been deleted in the abstract.**

**Comment:** Additionally, the results section of the abstract need improvement, as it does not adequately report the findings, and the percentage prevalence of child health deprivation mentioned is not found in the methodology.

**Response: Thank you for this observation. The result section of the abstract has been improved. Also, the percentage prevalence of child health deprivation has been provided.**

**Comment:** Lastly, the authors should discuss potential policy recommendations or future research directions based on the results, and since diarrhea and fever were not included as variables in the analysis, they should not be mentioned in the conclusion regarding treatment services.

**Response: Thank you for providing these insights. We have redrafted the section to establish a clearer focus. Diarrhea and fever has been dropped in the conclusion.**

**Introduction:**

**Reviewer:** The introduction provides a strong foundation for the research. It effectively highlights the prevalence of child poverty and its impact especially focusing on child development, both globally and in Nigeria. Furthermore, the use of previous statistical results helps emphasize the gravity of the problem, making the argument more compelling. The authors clearly define "child deprivation" and its various dimensions, which enhances understanding. The introduction is generally well-structured, transitioning smoothly from global to regional issues. However, there are a few suggestions for improvement;

**Response: Thank you for your assessment and recommendation.**

**Comment:** The introduction is overly lengthy and contains several wordy or repetitive sentences, likely for emphasis. Additionally, there are inconsistencies in the use of terms such as "child health deprivation," "child deprivation," "health deprivation," "children under 5," and "under-five children," which can confuse the reader. It is important to stick to one consistent term throughout the introduction for clarity.

**Response: Thank you for providing these insights. We have incorporated this suggestion throughout the manuscript. We used under-five children and child health deprivation throughout the paper.**

**Comment:** Condensing the information by paraphrasing or summarizing instead of quoting would also help streamline the text.

**Response: Thank you for this observation. We have incorporated your comments.**

**Comment:** The connection between child poverty and health deprivation needs to be further strengthened. Although this was briefly discussed in the abstract, it would be more effective to elaborate on it by citing specific studies or statistics that show the direct impact of poverty on child health outcomes.

**Response: Thank you. We have incorporated your comments. The connection have been strengthened.**

**Comment:** Additionally, there is inconsistency in the statistics reported regarding child poverty trends in line “4” and “6” where the authors wrote “a drop from 385 million in 2022” “to 333 million in 2022” appears to have an error. The second figure should likely refer to a different year, so this should be corrected for clarity.

**Response: Thank you. We have redrafted the section to establish a clearer focus.**

**Comment:** The statement about the reduction in child poverty from 50.2% to 43.4% is also somewhat repetitive, as the previous sentence already discusses trends. Consider merging these points into a single, concise statement to reduce redundancy and improve flow.

**Response: Thank you for this observation. The statement has been corrected to avoid repetition of the same statement.**

**Literature:**

**Comment:** The definitions provided for child deprivation by UNICEF and CFI are helpful but slightly broad, relying on immunization, treatment for diarrhea, and skilled birth attendance.

**Response: Thank you. We have redrafted the section to establish a clearer focus.**

**Comment:** However, the study lacks a nuanced discussion of broader definitions of child health deprivation.

**Response: Thank you for this observation. We have redrafted the section to establish a clearer focus.**

**Comment:** The second definition from CFI could benefit from a clearer transition between the three key concepts—deprivation, exclusion, and vulnerability—by streamlining the definitions and providing brief, clear explanations for each term. The authors should consider expanding the operationalization of child health deprivation by discussing it in terms of access to healthcare, nutrition, sanitation, or psychosocial well-being. For instance, WHO and UNICEF use a multidimensional approach to child deprivation that includes nutrition, education, and living conditions.

**Response: Thank you for this observation. We have redrafted the section to established a clearer focus**

**Comment:** The review briefly explain socio-economic factors, household size, and family characteristics but does not adequately discuss other important drivers, such as maternal education, geographic barriers to healthcare, and cultural factors.

**Response: Thank you for this interesting question. Maternal education, geographic barriers to healthcare, and cultural factors have been discussed.**

**Comment:** The authors should explore how parental education, access to maternal health services, cultural beliefs about health, and regional healthcare infrastructure contribute to child health deprivation.

**Response: Thank you. We have redrafted the section to establish a clearer focus**.

**Comment:** Additionally, the role of gender inequality in healthcare access, which disproportionately affects children, particularly girls, in some regions, should also be considered.

**Response: Thank you for this observation. We have redrafted the section to establish a clearer focus**.

**Comment:** While the review mentioned urbanization, it does not fully address the urban-rural health divide, especially in relation to urban poverty and slums, which significantly contribute to child health deprivation.

**Response: Thank you for this observation. We have incorporated your comments.**

**Comment:** The authors should expand on urban-rural disparities by citing literature on how urbanization exacerbates inequalities in healthcare access, particularly in rapidly growing slum populations.

**Response: Thank you for this observation. We have incorporated your comments.**

**Comment:** Studies from African countries could illustrate the nuances of urban poverty compared to rural deprivation, including the environmental risks in slum areas, such as pollution, poor sanitation, and unsafe drinking water, which need more thorough discussion.

**Response: Thank you for this observation. We have redrafted the section to established a clearer focus**

**Comment:** Although the review focuses on Nigeria, it misses the opportunity to place the country's child health deprivation in a global context.

**Response: Thank you for this observation. We have redrafted the section to established a clearer focus**

**Comment:** The authors should include comparative studies from other developing regions, such as South Asia or Latin America, facing similar challenges.

**Response: Thank you for this observation. We have redrafted the section to established a clearer focus**

**Comment:** They could discuss whether Nigeria's child health deprivation patterns mirror or diverge from those in other African countries or developing regions.

**Response: Thank you for this observation. We have redrafted the section to established a clearer focus**

**Comment:** Recent global reports from organizations like the World Bank, WHO, or UNICEF could provide valuable comparative data, framing Nigeria’s situation within broader global health trends.

**Response: Thank you for this observation. We have redrafted the section to establish a clearer focus.**

**Comment:** The transition from global data to Nigeria-specific information could be improved by adding a sentence or two that explicitly connects global trends in child health deprivation to Nigeria’s context before delving into local data.

**Response: Thank you for this observation. We have redrafted the section to established a clearer focus**

**Comment:** The introduction of the discussion on health insurance seems abrupt and would benefit from a clearer link to child health deprivation.

**Response: Thank you for this observation. We have redrafted the section to established a clearer focus**

**Comment:** The authors should first explain how healthcare access impacts child health deprivation before introducing the state of Nigeria’s health insurance system.

**Response: Thank you for this observation. We have redrafted the section to established a clearer focus**

**Comment:** Minor grammatical improvements, such as changing "present a need to probe" to "indicate the need to investigate," could enhance readability.

**Response: Thank you for this observation. We have redrafted the section to established a clearer focus**

**Comment:** Finally, although regional disparities in child health deprivation are mentioned, this important theme gets overshadowed by general discussions of deprivation. The concluding sentence is too long and lacks specificity about how the authors' findings will contribute to addressing child health deprivation. Clarifying these points would strengthen the conclusion.

**Response: Thank you for this observation. We have redrafted the section to established a clearer focus**

**Methodology:**

**Reviewer:** The methodology section is thorough, but there are a few areas that could benefit from clarification, restructuring, and some technical adjustments. Here are specific recommendations for improvement.

**Response: Thank you.**

**Comment:** The details about the MICS 2021 survey and its sampling frame are repeated in both the survey design and data source sections, which could be streamlined for clarity.

**Response: Thank you for this observation. Both data source and survey design has been streamline to avoid duplications of MICS 2021 survey and its sampling frame.**

**Comment:** There is confusion between the original sample size (31,103) and the final sample size (26,639) after weighting, but no explanation is given for this discrepancy.

**Response: Thank you for this observation. Inconsistency in the sample resulted from merging of data sets. The study used successful merged and weighted sample size for the study is 26,639**

**Comment:** Additionally, the methodology lacks mention of the software or statistical techniques used for data analysis.

**Response: Thank you. However, the software or statistical techniques used for this study was mentioned in “Statistical analysis”. (i. e. ‘Data were analysed using Stata SE Version 14, a statistical analysis program that incorporates the complex survey design as used in the MICS’**

**Comment:** While the description of the statistical techniques is thorough, it is somewhat verbose and could be more concise.

**Response: Thank you for this observation. The description of the statistical techniques has been corrected and detailed.**

**Comment:** The rationale for choosing specific statistical methods is not clearly explained.

**Response: Thank you for this observation. It has been earlier stated that the outcome variable (child deprivation) was dichotomous in nature thus, Binary Logistic Regression Model.**

**Comment:** Though the data cleaning process is mentioned, it lacks sufficient detail on the steps taken to check for errors.

**Response: Thank you for this observation. Because of the large number of primary sampling units (PSUs), Taylor Linearized methods was used to estimate standard errors for the coefficients of linear regression models fit to multi-stage samples.**

**Comment:** The results for the parameters of the binary logistic regression are not included, and the goodness-of-fit measure for the logistic regression model, derived from the likelihood ratio test, is missing. This measure is important as it indicates how well the model predicts the outcome variable.

**Response: Thank you for this observation. Parameters have been included.**

**Comment:** Consequently, the methods used to assess the overall performance of the fitted binary logistic regression model are also omitted.

**Response: Thank you for this observation. Details on fitted binary logistic regression model have been included.**

**Comment:** Streamlining these aspects and providing necessary details on data analysis would improve the clarity and completeness of the methodology.

**Response: Thank you for this observation. We have incorporated your comments.**

**Results:**

**Comment:** Tables 1, 2, 3, and 4 are missing.

**Response: Thank you. No. There was a mixed-up in the loading of the manuscript. We have two versions in the system. Table 1, 2, 3, and 4 are now presented in one file.**

**Comment:** As mentioned earlier in the preceding paragraph, some statistical results are not included.

**Response: Thank you for this observation. The missing statistical results in Table 4 are now included.**

**Comment:** It is also very important to check for the presence or absence of multicollinearity before analysing the data using binary logistic regression which is missing in this study.

**Response: Thank you for this observation.** A Multicollinearity test was performed using a variance inflation factor (VIF < 5). All explanatory variables with evidence of no collinearity (mean VIF= 1.38, maximum = 1.87 and minimum VIF = 1.10) were retained in the models while those with evidence of collinearity (VIF > 5) were excluded from the models

**Comment:** The results of the findings the authors presented does not capture the research questions. There is need for more statistical analysis that would further strengthen the research study.

**Response: Thank you for this observation. Binary logistic regression has been added.**

**Discussion:**

**Reviewer:** The discussion section of the study does a commendable job of linking the findings with previous literature, but there are a few gaps that could be addressed for a more comprehensive analysis. Below are the missing points and areas that could benefit from further elaboration

. **Response: Thank you**

**Comment:** While the authors acknowledge the limitations of using secondary data, they did not adequately discuss how this affects the validity or generalizability of the findings. For example, the results may be skewed due to underreporting in certain regions or among certain demographic groups? Providing a more detailed discussion of how secondary data limitations could have influenced key findings and offering suggestions for future studies using primary data would enhance the rigor of the analysis.

**Response: Thank you for this observation. However, one of the limitations that affects the generalization of research results is a small sample size. In this study, the sample size is large. In addition, truly some criteria or indicators may not be the same or quite fit the same frame or boundaries as collecting our own primary data. However, MICS is relevant, reliable or accurate for this study research objectives.**

**References**

**Comment:** The references appear to be well-written and relevant to the topic of child health deprivation in Nigeria. However, there seems to be a duplicate entry for the Nigeria Multiple Indicator Cluster Survey (MICS) (references 17 & 18). The authors might want to confirm if they are indeed different reports.

**Response: Thank you for this observation. They are not the same. Reference 17 was MICS 2021 while Reference 18 was MICS 2016-17. However, Reference 18 has been corrected to read:**

‘National Bureau of Statistics (NBS) and United Nations Children’s Fund (UNICEF). 2017 Multiple Indicator Cluster Survey 2016-17, Survey Findings Report. Abuja, Nigeria: National Bureau of Statistics and United Nations Children’s Fund. <https://www.unicef.org/nigeria/sites/unicef.org.nigeria/files/2018-09/Nigeria-MICS-2016-17.pdf>’

**Comment:** Also consider including geographically relevant resources e.g., while the focus is on Nigeria, some references on child health in neighbouring countries or similar contexts might be insightful for comparison.

**Response: Thank you. However, we have similar studies from Ghana, Kenya, etc. already cited in the references.**

Yours sincerely,

Dr. Joseph A. Kupoluyi

Demography and Social Statistics.
