# Supplementary material for: Regional variations in child health deprivation and its associated factors in Nigeria
Source: PLOS Glob Public Health. 2025 Aug 1;5(8):e0003796. doi: 10.1371/journal.pgph.0003796 (PMC12316245; doi:10.1371/journal.pgph.0003796)
Supplement: Attachment — Submitted filename: Response_to_Reviewers_auresp_2.docx [file pgph.0003796.s004.docx]

**Reviewer #3:**

The study investigates factors related to child health deprivation and how they vary across regions in Nigeria. Regions are defined in the study by geo-political zones. The study utilized the 2021 version of the Multiple Indicator Survey in Nigeria and found that basic and household characteristics were significant predictors of child health deprivation.

In all, the study is interesting, well-written and worthy of publication. However, there are fundamental issues that need to be addressed. I recommend a major revision of the manuscript before it is published by the Plos Global Public Health.

A fundamental issue with the manuscript is the lack of theoretical orientation or framework, and this is common among health scientists. The problem of regional variation, which the paper investigated, is a culturally and socially important one in Nigeria. It is a significant contribution that the study ought to make to scholarship. However, from the abstract to the conclusion, the issue was only discussed in statistical terms. Evidence in other studies shows that the north and south (and the geopolitical zones by extension) have significant socio-cultural differences, ultimately affecting health, economy and social activities. The study failed to adequately demonstrate why it is important to investigate regional differences in Nigeria. The academic world needs to know this! It is an important contribution to scholarship. I provide specific comments on each section of the manuscript.

Answer:	Thank you for your time and significant contributions to the manuscript. Your comments has been incorporated as follows:

**Abstract**

**Query:**	The introductory section of the abstract is a testament to the overall weakness of the manuscript. It says, “Nigeria has one of the worst child welfare metrics in the world, with child deprivation prevalent across all the social sectors. Children experience deprivation and poverty differently when compared to adults, and this ultimately affects their development. This study examined regional variations in child health deprivation and its associated factors in Nigeria.” Meanwhile, the issue of regional variations and its importance in Nigeria was not emphasized in the introduction but only in the results.

**Answer:**	Thank you for your observations and important contributions to this manuscript. Abstract introduction has been corrected to read ‘Child health deprivations differ by socio-cultural differences and some demographic and socio-economic factors. Deprivation may be more pronounced by the geo-political zones/regions in Nigeria given the differences in their socio-cultural, education, religion, and economic particularly between the North and the South geo-political zones. Thus, this study examined regional variations in child health deprivation and its associated factors in Nigeria. In addition, the reason for regional variation has been included in the abstract, body of the work, and in the theoretical section of the manuscript.

**Background**

**Query:** The background provides adequate information about the different forms of child deprivation and emphasizes child health deprivation in particular. However, the section did not do justice to the existence and importance of investigating regional differences in Nigeria. The mention of regional disparities in health and rural residence is not enough. The study claimed that “These studies somewhat focused on single indicators to measure child health deprivations or limited in its approach to examine regional variations by associated factors (p. 7)” but it also failed to explain why the academic world should care about regional variations in Nigeria. I have conducted research and collected primary data across geopolitical zones in Nigeria, and I encourage the authors to emphasize the socio-cultural differences across regions and geopolitical zones in the country, and how they affect health outcomes and consequently child health deprivation.

Since the introduction already appears too long, I encourage the authors to create a section for theoretical framework or “Importance of investigating regional variations in Nigeria.” My colleague in Nigeria has done some work in regional variations in Nigeria. The authors may utilize some of them if they find them necessary and relevant to their work.

<https://doi.org/10.1017/S0021932023000238>
<https://doi.org/10.1017/S0021932022000463>
<https://doi.org/10.1017/S0021932020000747>

That a manuscript is empirical does not mean it should be a theoretical. The authors may need to add that their study contributes to Sustainable Development Goal 10- Reduced inequalities.

**Answer:** Thank you for providing these insight. The importance of investigating regional differences in child health deprivation in Nigeria has been included in the manuscript. A section for the theoretical framework has been created to explaining the regional variations between the North and the South in Nigeria. Thank you for providing the articles links. They are of great help to us. The socio-cultural differences across regions and geopolitical zones in Nigeria as well as their effects in health outcomes and child health deprivation in Nigeria has been explored.

**Methods**

**Query:**	The authors need to re-write how they operationalized the dependent variable- child health deprivation. They should tell the readers about the scoring system. It is not enough to say the composite measures were converted to binary outcomes. How were the four variables scored? Is it ‘0’ and ‘1’ for each of them? If yes, what score range was used to determine “child-deprived” and “child-not deprived”? The authors wrote that “The composite variable was computed into a binary dimension of child-deprived (those who were not covered by full immunization, did not receive diarrhea treatment, fever treatment, and were not covered by the health insurance scheme) and child not deprived (those who were covered by immunization, have received diarrhea treatment from the health facility, received fever treatment, and covered by the health insurance scheme)”. There were four indicators:
• full immunization
• diarrhea treatment
• Fever treatment
• Health insurance

**Answer**:	Thank you. We agree with you and have incorporated how we operationalized the dependent variable- child health deprivation in the methods section of the manuscript. It now reads:

**“**Four variables were considered for operationalization of child health deprivation; (i) third dose of pentavalent vaccine (penta 3); (ii) care seeking at a health facility or provider for diarrhea; (iii) care seeking at a health facility or provider for fever; (iv) and health insurance coverage (children under-five). Response of receiving the service for each question was coded as ‘1= Yes’ and ‘0 = No’ otherwise. In order to generating a single, representative value for the outcome variable, deprivation for under-five children was measured as a composite of these four variables with under-five children who did not receive these services considered as health deprived and scored as ‘1’ and ‘0’ otherwise. Hence, a child is health deprived and scored as ‘1’ if; (i) the child did not receive the third dose of pentavalent vaccine, (ii) the child did not receive treatment or advice for diarrhoea from a health facility or provider, (iii) the child did not receive treatment or advice for fever, and (iv) the child did not covered by any health insurance, and otherwise ‘0’. Then, we summed the resulting scores of all the four indicators ranging from 0 to 4. Therefore, a child is considered to be health deprived if two or more of the condition stated is achieved. Thus, those who were not covered by full immunization, did not receive diarrhea treatment, fever treatment, and were not covered by the health insurance scheme were coded as ‘1’ and ‘0’ otherwise (i.e. those who were covered by immunization, have received diarrhea treatment from the health facility, received fever treatment, and covered by the health insurance scheme) [18, 19]. A Cronbach’s alphas of 0.80 reliability analysis on the composite score implies that the combined items has acceptable reliability”.

**Query:** How did you categorise those who had two or three out of these four? If my child had full immunization, received diarrhea treatment and fever treatment but did not have health insurance, would you say (s)he is deprived?

**Answer**:	Thank you for this question. No. (s)he is not deprived. Deprivation was based on having two or more of these four indicators.

**Query:**	The authors wrote “The geopolitical zones were the main explanatory variable, and it was measured as zones.” Again, this explains why regional differences need to be grounded theoretically in the work. The authors need to link the relevance of “other explanatory variables” and “intervening variables” to the study. In large datasets, associational tests are likely to be statistically significant. The authors need to state how other variables logically relate to the main (in) dependent variable. The section on “statistical analyses” can be discussed at descriptive and inferential for the sake of coherence.

**Answer:** Thank you for your suggestion. We have incorporated this suggestion into the statistical analyses sub-section.

**Query:** I respect your choice of statistics and analyses, but have you ever come across situations where variables are insignificant at a bivariate level but appear statistically significant in multivariate analyses?

**Answer**: Thank you. You are right. Bivariate insignificant variable can turn up statistical significant in a multiple regression. That is, insignificant variables may become significant or even change their direction of effect (e.g., from negative to positive) due to the interaction and correlation with other variables in the model. This phenomenon is often referred to as a "suppressor effect" or "Simpson's paradox. Nevertheless, including Bivariate insignificant variables can also obscure their true relationship. Thus, this study exclude them. Future studies can also investigate their inclusion.

**Results**

**Query:**	The authors should show the mean and SD of the ratio variables (age, household size, number of children, etc).

**Answer**:	Thank you for your suggestion. We have incorporated this suggestion by adding the mean and standard deviation to the suggested variables (i.e. age of household head, household size, number of children).

**Query:**	The label of the figures should be at the bottom, not inside the figures.

**Answer**:	Thank you. We have removed the label of the figures (Figure 1 and 2) to the bottom of the figures.

**Query:**	Interestingly, the question I asked in the method popped up again in Table 1. Health insurance issues are complex in Nigeria; again, if my child had “yes” in the three other variables but “no” for health insurance, does that mean (s)he is deprived, considering the structure and nature of health insurance in Nigeria? The context of the location of your research matters!

SINCE 97% OF THE CHILDREN ARE DEPRIVED, ACCORDING TO THE TREATMENT OF YOUR COMPOSITE MEASURES, THEN WHAT IS THE BASIS FOR ASSOCIATIONAL ANALYSIS?

**Answer**:	Thank you. We agreed with you that the structure and nature of health insurance in Nigeria is a complex issues. This is reflected in Figure 1 as suggested however, deprivation in this study was based on having two or more of these four indicators particularly full immunization, diarrhea and treatment for fever.
