# Supplementary material for: Regional variations in child health deprivation and its associated factors in Nigeria
Source: PLOS Glob Public Health. 2025 Aug 1;5(8):e0003796. doi: 10.1371/journal.pgph.0003796 (PMC12316245; doi:10.1371/journal.pgph.0003796)
Supplement: Attachment — Submitted filename: Response_to_Reviewers_auresp_3.docx [file pgph.0003796.s005.docx]

Jun 28 2025 11:59PM

Journal Requirements:

**Comment:** Please review your reference list to ensure that it is complete and correct. If you have cited papers that have been retracted, please include the rationale for doing so in the manuscript text, or remove these references and replace them with relevant current references. Any changes to the reference list should be mentioned in the rebuttal letter that accompanies your revised manuscript. If you need to cite a retracted article, indicate the article’s retracted status in the References list and also include a citation and full reference for the retraction notice.

**Answer:** Thank you. Reference has been checked.

Reviewer #3:

**Comment**: Thank you for submitting your manuscript to PLOS Global Public Health. After careful consideration, we feel that it has merit but does not fully meet PLOS Global Public Health’s publication criteria as it currently stands. Therefore, we invite you to submit a revised version of the manuscript that addresses the points raised during the review process.
Some further minor revisions have been requested: Reviewer 3 requested further discussion of the extent to which the assumptions of the CSCFH support/contradict the results of your study needs to be discussed in your results and discussion of findings. Which region has made progress and which has failed as far as child deprivation is concerned? To what extent is this a function of the region's socio-cultural and economic outlook and beliefs?

**Answer:** Thank you. The CSCFH has been incorporated into the discussion.

**Comment:** While revising your submission, please upload your figure files to the Pre-flight Analysis and Conversion Engine (PACE) digital diagnostic tool, https://pacev2.apexcovantage.com/. PACE helps ensure that figures meet PLOS requirements. To use PACE, you must first register as a user. Registration is free. Then, login and navigate to the UPLOAD tab, where you will find detailed instructions on how to use the tool. If you encounter any issues or have any questions when using PACE, please email PLOS at figures@plos.org. Please note that Supporting Information files do not need this step.

Answer: Thank you. Figures have been uploaded to meet PLOS requirements.
