# Supplementary material for: Regional variations in child health deprivation and its associated factors in Nigeria
Source: PLOS Glob Public Health. 2025 Aug 1;5(8):e0003796. doi: 10.1371/journal.pgph.0003796 (PMC12316245; doi:10.1371/journal.pgph.0003796)
Supplement: Attachment — Submitted filename: Response_to_Reviewers_auresp_4.docx [file pgph.0003796.s006.docx]

10 of 3,451

Reviewer #3: Dear author,

**Comment:** Thank you for submitting the revised version of your manuscript. I looked at the different versions of the manuscript, including the original version and revisions 1, 2 and 3. Below are my comments to your previous version: “The operationalization of outcome variables is now clearly presented. The conceptual framework upon which the work rests is clear. However, the conceptual/theoretical orientation is hanging. It was only discussed in the beginning part of the work (pages 7-9). The extent to which the assumptions of the CSCFH support/contradict the results of your study needs to be discussed in your results and discussion of findings. Which region has made progress and which has failed as far as child deprivation is concerned? To what extent is this a function of the region's socio-cultural and economic outlook and beliefs?” That was my honest and sincere evaluation of your revised manuscript. I acknowledged that the theoretical framework was clear. All you needed to do was to integrate it into the discussion section. However, you have completely removed the section on theoretical framework titled “Rationale for investigating regional variations” and only discuss the regional issues briefly. This makes the work unscholarly. Now, CSCFH has been stated without reference to the origin of the theory or earlier works therein. I am not sure if this was deliberate or an oversight. The R2 version of the manuscript read even better than the current version. I understand that this is your manuscript, but I must sincerely say that the current version is unscholarly. The title of your work is “Regional variations in child health deprivation and its associated factors in Nigeria.” That particular section on “rationale for investigating regional variations in Nigeria” gave theoretical and conceptual weight to your study, but you have removed it and made it look like CSCFH is original to you. This is not good scholarship!. I see you have integrated the CSCFH into the discussion, but what sense does it make now when the section on theoretical framework has been removed and only briefly mentioned as part of the introduction? You need to bring back the theoretical framework as it was in the previous version PGPH-D-24-01755R2 and leave the current discussion as is.

**Answer:** What! Oh my God! Thank you for this great observation. It was not intentional. I mistakenly worked on the wrong manuscript file. Sorry for this. For now, the correct manuscript has been updated with the theoretical framework. The current discussion as at last reviewed with CSCFH has been retained. Thank you.
